# Supplementary material for: Morpho-Cultural and Pathogenic Variability of Sclerotinia sclerotiorum Causing White Mold of Common Beans in Temperate Climate
Source: J Fungi (Basel). 2022 Jul 21;8(7):755. doi: 10.3390/jof8070755 (PMC9316490; doi:10.3390/jof8070755)
Supplement: Supplementary file 1 [file jof-08-00755-s001.zip › jof-1794401-supplementary.pdf]

SUPPLEMENTARY MATERIAL

Table S1. Analysis of variance table for two factorial analyses of Disease reactions of six isolates of S. sclerotiorum and eighteen bean genotypes.

Source of Variation	DF	Sum of Squares	Mean Squares	F-Cal	Sig.
Lines	17	627.247	36.897	20.900	0.00
Isolates	5	172.617	34.523	19.555	0.00
Interaction Lines X Isolates	85	497.383	5.852	3.315	0.00
Error	216	381.333	1.765		
Total	323	1678.580			
